# An Antibiotic Prophylaxis for Prevention of Ventriculoperitoneal Shunt Infection Using Intraventricular Injection and Shunt Soaking with Vancomycin and Gentamicin

**DOI:** 10.3390/antibiotics15010060

**Published:** 2026-01-05

**Authors:** Saruta Khunchamnan, Intouch Sopchokchai, Kittisak Sawanyawisuth, Amnat Kitkhuandee

**Affiliations:** 1 Department of Surgery and Medicine, Faculty of Medicine, Khon Kaen University, Khon Kaen 40002, Thailand; namkhing.saruta@gmail.com (S.K.); kittisak@kku.ac.th (K.S.); 2 Department of Neurosurgery, Neurological Institute of Thailand, Bangkok 10400, Thailand; i_sopchokchai@hotmail.com

**Keywords:** vancomycin, gentamicin, age

## Abstract

**Background/Objectives:** There is limited evidence of a combination of intraventricular injection and shunt soaking with a vancomycin–gentamicin technique as a prophylaxis for shunt infection. This study aimed to evaluate if a combination of this prophylaxis technique was a potential strategy in preventing ventriculoperitoneal (VP) shunt infection. Factors associated with VP shunt infection at one year were executed by using logistic regression analysis. **Methods:** This was a retrospective cohort study. The inclusion criteria were consecutive patients who received VP shunt placement regardless of etiology. The primary outcome of this study was VP shunt infection at one year postoperatively. **Results:** During the study period, there were 413 patients who met the study criteria. Of those, 31 patients (7.51%) had an infected VP shunt one year after the operation. There were three factors that were independently associated with VP shunt infection at one year: age, etiology of brain tumor, and intraventricular injection and shunt soaking technique. The adjusted odds ratio of age and brain tumor was 0.974 (95% confidence interval of 0.960, 0.986) and 0.251 (95% confidence interval of 0.099, 0.640), while intraventricular injection and shunt soaking technique had an adjusted odds ratio of 0.422 (95% confidence interval of 0.212, 0.768). **Conclusions:** A combination of intraventricular injection and shunt soaking technique with vancomycin and gentamicin may lower the VP shunt infection rate at one year after operation. Younger patients under an age of 8 years may be at risk for VP shunt infection. Further prospective randomized controlled trial may be needed to confirm the results of this study.

## 1. Introduction

The ventriculoperitoneal shunt (VP shunt) is a treatment for hydrocephalus and normal pressure hydrocephalus. A previous study conducted in 1173 patients showed that the infection rate of the shunt was 13.6% [1]. Several risk factors for shunt infection were reported, including younger age below 4 months with an odds ratio of 1.81 (*p* = 0.011) or antenatal diagnosis with an odds ratio of 2.23 (*p* = 0.032). The overall mortality rate of shunt infection was 10.1%. Effective prevention strategies are required to prevent shunt infection postoperatively.

The BASICS trial compared three interventions (silver-impregnated, antibiotic-impregnated, or standard) to prevent VP shunt infection [2]. Only the antibiotic-impregnated arm lowered shunt infection by 62% (*p* = 0.0038). Two antibiotics, rifampicin and clindamycin, were used in the BASICS trial. As Staphylococcus aureus or epidermidis were the two most common pathogens in VP shunt infection, up to 60% [3,4], intraventricular vancomycin significantly reduced VP shunt infection than the control (3.2% vs. 6.9%; *p* = 0.03) [4]. Another study found that antibiotic reservoir injection had a significantly lowered shunt infection rate than the control (2.6% vs. 6.3%; *p* = 0.0455) [5]. There is limited evidence on a combination of intraventricular injection and shunt soaking with the vancomycin–gentamicin technique as a prophylaxis for shunt infection. This study aimed to evaluate if a combination of this prophylaxis technique was a potential strategy in preventing VP shunt infection.

## 2. Materials and Methods

This was a retrospective cohort study conducted at Khon Kaen University Hospital, Thailand. The inclusion criteria were consecutive patients who received VP shunt placement regardless of etiology. Those who received multiple VP shunt placements were excluded. The study period was between October 2014 and September 2018. Eligible patients were evaluated for baseline characteristics, surgical factors, and the outcome: VP shunt infection at one year after the placement. Baseline characteristics included age, sex, etiology of VP shunt placement, underlying diseases, previous history of shunt infection, and previous abdominal surgery. Surgical factors were surgical time, type of shunt (fixed pressure or programmable), CSF diversion [6], and intraventricular injection and shunt soaking technique.

The intraventricular injection and shunt soaking technique was performed since February 2017 with evidence from previous studies [5,7]. This antibiotic prophylaxis was performed in consecutive cases requiring a VP shunt regardless of surgeons, patient characteristics, or clinical urgency unless contraindicated by a history of antibiotic allergy (Table 1). There were two parts of the antibiotic prophylaxis: intraventricular antibiotic injection and VP shunt soaking with antibiotics. The intraventricular antibiotic injection composed of 4 mg of gentamicin and 10 mg of vancomycin injection into intraventricular space via proximal catheter before connecting the system, while VP shunt soaking was performed by using 76 mg of gentamicin and 490 mg of vancomycin, which were diluted in normal saline 100 mL for soaking all shunt components including proximal catheter, reservoir, and distal catheter. The solution was manually pumped into the reservoir of the shunt until fully filled. The total dose of gentamicin was 80 mg and 500 mg of vancomycin. Both intraventricular antibiotic injection and VP shunt soaking with antibiotics were performed separately. Another intravenous antibiotic prophylaxis was 1 g of cefazolin or clindamycin 10 mg/kg in patients with penicillin allergy. The intravenous antibiotic prophylaxis was administered 24 h postoperatively.

The primary outcome of this study was VP shunt infection at one year postoperatively. The VP shunt infection was defined by the presence of one of the following criteria: positive CSF culture or catheter tip culture; symptoms and/or signs compatible with shunt infection, i.e., fever, headache, meningitis, peritonitis, CSF pleocytosis with predominance of polymorphonuclear leukocytes, or low CSF glucose; or localized findings consistent with shunt infection such as abdominal pseudocyst, inflammation along the shunt tract, or shunt erosion.

In the statistical analysis, patients were categorized into two groups based on the primary outcome: infected VP shunt and non-infected VP shunt. Numerical studied variables were reported as median (interquartile range), while categorical studied variables were reported as number (percentage). Differences in studied variables between both groups were computed by using Wilcoxon rank sum test for numerical studied variables and Fisher’s exact test for categorical studied variables. Additionally, clinical factors between those who received and did not receive the antibiotic prophylaxis were also computed.

Factors associated with VP shunt infection at one year were executed by using stepwise logistic regression analysis. Studied variables were calculated for a *p* value by using univariable logistic regression analysis. Factors with a *p* value of less than 0.20 by univariable logistic regression analysis or those potential variables for VP shunt infection were included in the multivariable logistic regression analysis. The predictive model for VP shunt infection was evaluated for goodness of fit by the Hosmer–Lemeshow method. A receiver operating characteristic (ROC) curve was computed for a significant numerical predictor for an infected VP shunt. An appropriate cut point was chosen to provide a sensitivity of 80% or more. Statistical analyses were computed by STATA software version 18.5 (College Station, TX, USA).

## 3. Results

During the study period, there were 413 patients who met the study criteria. Of those, 31 patients (7.51%) had an infected VP shunt one year after the operation. Regarding baseline characteristics (Table 2), there were three significant factors between those who had and did not have VP shunt infection in one year, including age, etiology, and previous shunt infection. The infected VP shunt group had a significantly young age (3 vs. 47 years; *p* < 0.001), higher proportion of patients with congenital brain anomalies (41.94% vs. 12.04%; *p* < 0.001), and higher proportion of previous VP shunt infection (9.68% vs. 2.88%; *p* = 0.044). There was no mortality during the study period as well as serious adverse events from the prophylaxis antibiotics including acute renal failure, auditory toxicity, or neurological toxicity.

There were comparable surgical factors between those who had and did not have a VP shunt infection at one year including surgical time, type of shunt, CSF diversion, and intraventricular injection and shunt soaking technique (Table 3). The infected VP shunt group had a lower proportion of intraventricular injection and shunt soaking technique than the non-infected VP shunt group (32.26% vs. 48.69%; *p* = 0.078). A predictive model for VP shunt infection at one year is shown in Table 4. There were three factors in the model. These three factors were independently associated with VP shunt infection at one year: age, etiology of brain tumor, and intraventricular injection and shunt soaking technique. The adjusted odds ratio of age and brain tumor was 0.974 (95% confidence interval of 0.960, 0.986) and 0.251 (95% confidence interval of 0.099, 0.640), while intraventricular injection and shunt soaking technique had an adjusted odds ratio of 0.422 (95% confidence interval of 0.212, 0.768). The model had goodness of fit with the Hosmer–Lemeshow Chi square of 8.87 (*p* = 0.354).

**Figure 1:**
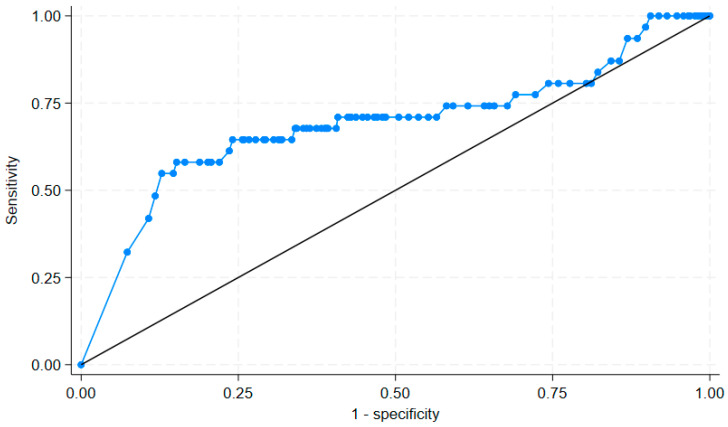
A receiver operating characteristic (ROC) curve of age to predict presence of ventriculoperitoneal shunt infection at one year after operation. The area under the ROC curve was 68.95%.

The area under the ROC curve for age to predict infected VP shunt was 68.95% (95% confidence interval of 57.08%, 80.80%), as shown in Figure 1. An age of 8 years or over had a sensitivity of 81.15% and specificity of 58.06% to predict uninfected VP shunt.

Clinical factors between those who received and did not receive intraventricular injection and shunt soaking were compared to evaluate selection bias. There were four significant factors between these two groups including proportions of hypertension, HIV infection, type of abdominal surgery wound, and type of shunt (Table 5). The intraventricular injection and shunt soaking had more patients with hypertension than the no treatment group (29.08% vs. 20.28%; *p* = 0.040), the other four factors were found to be higher proportions in the no treatment group. There was no significant difference regarding pathogens of infected VP shunt between both groups.

## 4. Discussion

This study found that the overall VP shunt infection rate was 7.51%, which was slightly lower than the previous from Korea [8]. The VP shunt infection rate in the Korean study was 10.5%. These differences may be explained by two factors: age group of study patients and antibiotic treatment. The previous Korean study was conducted in 224 children with antibiotic prophylaxis by first generation cephalosporin (81.4%) and vancomycin (10.8%). As previously reported, younger age was a main risk factor for VP shunt infection [8,9,10]. Children under 1 year had a relative risk of VP shunt infection at 2.31 (95% confidence interval of 1.19, 4.48) [8]. Similarly, this study found that an increasing age had lower VP shunt infection rate with an adjusted odds ratio of 0.978 (95% confidence interval of 0.961, 0.995), as shown in Table 3. A cut point of age at 8 years or older was shown to be a lower risk of VP shunt infection in this study. Brain tumor negative association with VP shunt infection: adjusted odds ratio of 0.251 (Table 4). A previous meta-analysis also reported this similar finding with an odds ratio of 0.418 (95% confidence interval of 0.256, 0.682) [11].

Intraventricular injection and shunt soaking technique was another independent protector for VP shunt infection. The VP shunt infection rate was lower in those who received intraventricular injection and shunt soaking technique than those without (5.10% vs. 9.68%; *p* = 0.078), as shown in Table 2. The intraventricular injection and shunt soaking technique showed a lower infection rate than the previous study at one year (5.10% vs. 10.5%) [8]. As mentioned earlier, the Korean study used perioperative antibiotics with first generation cephalosporin in the majority of cases which may have lower efficacy. Another study using intravenous ceftriaxone at induction of anesthesia plus 30 mL of gentamicin for VP shunt also had higher VP shunt infection rate at 7.2% [12]. These results may imply that the intraventricular injection and shunt soaking technique with gentamicin and vancomycin may have a lower VP shunt infection rate. Additionally, the effect of this antibiotic prophylaxis lasted for one year, as shown by this study. A previous study supported using antibiotic-impregnated shunt as it lowered the shunt infection rate by 2.8% [13]. Previous studies also showed that vancomycin and gentamicin had a low VP shunt infection rate, while cloxacillin had an adjusted hazard ratio of 3.83 (*p* = 0.001) for VP shunt infection [14,15]. These data supported the use of vancomycin and gentamicin for long-term VP shunt infection prophylaxis.

Regarding identification of pathogens of the VP shunt infection, 22 pathogens were identified by positive culture in 31 patients (70.97%). As previously reported, Staphylococcus aureus was the most common pathogen [16,17]. In children, Acinetobacter baumannii may be the common pathogen [18]. In this study, A. baumannii was found in two patients (9.09%). We also found that other Gram-negative bacteria and Candida were the possible pathogens (Table 5). Note that patients who received intra-ventricular injection and shunt soaking had a lower rate of S. aureus infection than the no treatment group, as one of the antibiotic regimens was vancomycin. 

The strength of this study was that the intraventricular injection and shunt soaking technique was a potential strategy in lowering VP shunt infection at one year. This technique may be suitable for those resource-limited facilities, particularly in developing countries, as it is a cheaper technique than impregnated shunt. Even though this was not a randomized controlled trial, most clinical factors in the treatment and no treatment group were comparable (Table 5). The five significant factors may be less likely to affect the infection outcome.

There are some limitations in this study. A study population comprising both children and adult patients receiving a VP shunt. Some factors were not studied including ICU stay, duration of external drains, presence of CSF leak, wound complications, length of hospital, or antibiotic susceptibility of the pathogens of infected VP shunt. Even though previous shunt infection was significant for VP shunt infection by inferential analysis, it was not significant by logistic regression analysis. Note that numbers of patients with an infected VP shunt was low. Second, the results of this study were specific to the combination of vancomycin and gentamicin intraventricular injection plus shunt soaking in a fixed dose fashion. Finally, the outcome was evaluated at one year, and was not classified as early or late and no other outcomes were evaluated, such as revised shunt. As this was a retrospective cohort study in a single institution, further prospective randomized controlled trial may be required particularly in other countries, different populations, and different age distributions.

## 5. Conclusions

A combination of intraventricular injection and shunt soaking technique with vancomycin and gentamicin may lower VP shunt infection rate at one year after operation. Younger patients under an age of 8 years may be at risk for VP shunt infection. Further, a prospective randomized controlled trial may be needed to confirm the results of this study.

## Figures and Tables

**Table 1:** Patients received a ventriculoperitoneal shunt placement received antibiotic prophylaxis with intraventricular injection and shunt soaking by year.

Year	No Antibiotic Prophylaxis	Antibiotic Prophylaxis	Total
2014	13	0	13
2015	90	0	90
2016	105	2	107
2017	9 *	124	133
2018	0	69	69
2019	0	1	1

Note. Data shown as number of patients; * VP shunt was performed prior to February 2017.

**Table 2:** Baseline characteristics of patients received a ventriculoperitoneal shunt placement categorized by infection of shunt at one year.

Factors	Non-Infected *n* = 382	Infected *n* = 31	*p*-Value
Median (IQR) age, years	47 (13–62)	3 (0–59)	<0.001
Male sex	181 (47.38)	19 (61.26)	0.136
Etiology			
Meningitis	29 (7.59)	0	0.151
Intracerebral hemorrhage	109 (28.53)	10 (32.26)	0.682
Brain tumor	173 (45.29)	6 (19.35)	0.005
Congenital	46 (12.04)	13 (41.94)	<0.001
Others	25 (6.54)	2 (6.45)	0.999
Underlying diseases	53 (13.87)	4 (13.33)	0.934
Hypertension	97 (25.39)	4 (12.90)	0.120
Dyslipidemia	17 (4.45)	2 (6.45)	0.609
Stroke	16 (4.19)	0 (0.00)	0.245
Cancer	19 (4.97)	1 (3.23)	0.663
Adrenal insufficiency	14 (3.66)	0 (0.00)	0.278
Chronic kidney disease	6 (1.57)	0 (0.00)	0.482
Seizure	4 (1.05)	1 (3.23)	0.286
HIV infection	7 (1.83)	0 (0.00)	0.447
Hypothyroidism	10 (2.62)	0 (0.00)	0.362
Previous shunt infection	11 (2.88)	3 (9.68)	0.044
Previous abdominal surgery	7 (1.83)	1 (3.23)	0.588
Type of wound			0.424
Clean	4 (1.05)	1 (3.23)	
Clean-contaminated	2 (0.52)	0	

Note. Data presented as number (percentage) unless indicated otherwise; IQR: 1st–3rd interquartile range.

**Table 3:** Surgical factors of patients received a ventriculoperitoneal shunt placement categorized by infection of shunt at one year.

Factors	Non-Infected *n* = 382	Infected *n* = 31	*p*-Value
Median (IQR) surgical time, minute	60 (45–71)	52 (45–65)	0.215
Type of shunt			0.613
Fixed pressure shunt	368 (96.34)	31 (100)	
Programmable shunt	14 (3.66)	0	
CSF diversion	306 (80.10)	22 (73.33)	0.375
Intraventricular injection and shunt soaking	186 (48.69)	10 (32.26)	0.078

Note. Data presented as number (percentage) unless indicated otherwise; IQR: 1st–3rd interquartile range; CSF: cerebrospinal fluid.

**Table 4:** Factors associated with ventriculoperitoneal shunt infections at one year after the operation.

Factors	Unadjusted Odds Ratio (95% Confidence Interval)	Adjusted Odds Ratio (95% Confidence Interval)
Age	0.973 (0.958, 0.988)	0.974 (0.960, 0.986)
Etiology: Brain tumor	0.290 (0.116, 0.722)	0.251 (0.099, 0.640)
Intraventricular injection and shunt soaking	0.502 (0.230, 1.093)	0.422 (0.212, 0.768)

Note. Factors included in the stepwise model were congenital etiology and surgical time.

**Table 5:** Baseline characteristics of patients received a ventriculoperitoneal shunt placement categorized by treatment with intraventricular injection and shunt soaking.

Factors	No Treatment *n* = 217	Treatment *n* = 196	*p*-Value
Median (IQR) age, years	43 (10–60)	48 (13–64)	0.536
Male sex	121 (55.76)	92 (46.94)	0.077
Etiology			
Meningitis	17 (7.83)	12 (6.12)	0.566
Intracerebral hemorrhage	54 (24.88)	65 (33.16)	0.066
Brain tumor	104 (47.93)	75 (38.27)	0.059
Congenital	28 (12.90)	31 (15.82)	0.403
Others	14 (6.45)	13 (6.63)	0.999
Underlying diseases			
Hypertension	44 (20.28)	57 (29.08)	0.040
Dyslipidemia	11 (5.07)	8 (4.08)	0.815
Stroke	5 (2.30)	11 (5.61)	0.123
Cancer	8 (3.69)	12 (6.12)	0.262
Adrenal insufficiency	8 (3.69)	6 (3.06)	0.791
Chronic kidney disease	2 (0.92)	4 (2.04)	0.429
Seizure	3 (1.38)	2 (1.02)	0.999
HIV infection	7 (3.23)	0	0.016
Hypothyroidism	5 (2.30)	5 (2.55)	0.999
Previous shunt infection	9 (4.15)	5 (2.55)	0.425
Previous abdominal surgery	7 (3.23)	1 (0.51)	0.070
Type of wound			0.031
Clean	5 (2.30)	0	
Clean-contaminated	2 (0.92)	0	
Median (IQR) surgical time, minute	60 (40–70)	60 (45–71)	0.215
Type of shunt			0.005
Fixed pressure shunt	215 (99.08)	184 (93.88)	
Programmable shunt	2 (0.92)	12 (6.12)	
CSF diversion	177 (81.57)	151 (77.44)	0.328
Pathogens	12 (5.53)	10 (5.10)	0.999
Coagulase negative *Staphylococcus*	9	4	
*Pseudomonas* spp.	2	1	
*Acinetobacter baumannii*	0	2	
*Klebsiella pneumoniae*	1	1	
*Bacillus* spp.	0	1	
*Candida albicans*	0	1	

Note. Data presented as number (percentage) unless indicated otherwise; IQR: 1st–3rd interquartile range.

## Data Availability

The original contributions presented in this study are included in the article. Further inquiries can be directed to the corresponding author.

## References

[1] Vinchon M., Dhellemmes P. (2006). Cerebrospinal Fluid Shunt Infection: Risk Factors and Long-Term Follow-Up. Child’s Nerv. Syst..

[2] Mallucci C.L., Jenkinson M.D., Conroy E.J., Hartley J.C., Brown M., Dalton J., Kearns T., Moitt T., Griffiths M.J., Culeddu G. (2019). Antibiotic or Silver versus Standard Ventriculoperitoneal Shunts (BASICS): A Multicentre, Single-Blinded, Randomised Trial and Economic Evaluation. Lancet.

[3] Campbell D., Sinclair S., Cooke D., Webster D., Reid M. (2023). The Incidence of VP Shunt Infection in a Middle-Income Nation: A Retrospective Analysis of a Pediatric Population. Front. Surg..

[4] Raygor K.P., Oh T., Hwang J.Y., Phelps R.R.L., Ghoussaini K., Wong P., Silvers R., Ostling L.R., Sun P.P. (2020). Ventriculoperitoneal Shunt Infection Rates Using a Standard Surgical Technique, Including Topical and Intraventricular Vancomycin: The Children’s Hospital Oakland Experience. J. Neurosurg. Pediatr..

[5] Burrows A.M., Murphy M.E., Daniels D.J., Meyer F.B. (2016). Antibiotic Reservoir Injection Reduces Shunt Infection in Adults. World Neurosurg..

[6] Stewart J., Warner-Levy J., Bate S., McMahon C., Slade D., Bailey M. (2025). Determinants of Adult Ventriculoperitoneal Shunt Failure: Insights from a Large Neurosurgical Centre. Clin. Neurol. Neurosurg..

[7] Ragel B.T., Browd S.R., Schmidt R.H. (2006). Surgical Shunt Infection: Significant Reduction When Using Intraventricular and Systemic Antibiotic Agents. J. Neurosurg..

[8] Lee J.K., Seok J.Y., Lee J.H., Choi E.H., Phi J.H., Kim S.-K., Wang K.-C., Lee H.J. (2012). Incidence and Risk Factors of Ventriculoperitoneal Shunt Infections in Children: A Study of 333 Consecutive Shunts in 6 Years. J. Korean Med. Sci..

[9] Bastian R.A., Pramusinto H., Basuki E., Marianne M. (2022). Ventriculoperitoneal Shunt Infection: A Study about Age as a Risk Factor in Hydrocephalus Pediatrics. Open Access Maced. J. Med. Sci..

[10] Agarwal N., Shukla R.M., Agarwal D., Gupta K., Luthra R., Gupta J., Jain S. (2017). Pediatric Ventriculoperitoneal Shunts and Their Complications: An Analysis. J. Indian Assoc. Pediatr. Surg..

[11] Signorelli F., Palermo M., Onorati F., Zeoli F., Romozzi M., Marziali G., Sturiale C.L., Trevisi G., Visocchi M. (2025). Risk Factors for Ventriculoperitoneal Shunt Infection: A Systematic Review and Meta-Analysis. Brain Sci..

[12] Ayogu O.M., Igbokwe K.K., Jabir K.M., Onobun E.D., Okpata C.I., Ugwuanyi U., Ekpendu I., Essiet E.A. (2024). Ventriculoperitoneal Shunt Infection Rate and Other Associated Complications of VP Shunt Insertion in Abuja, Nigeria. World Neurosurg. X.

[13] Farber S.H., Parker S.L., Adogwa O., McGirt M.J., Rigamonti D. (2011). Effect of Antibiotic-Impregnated Shunts on Infection Rate in Adult Hydrocephalus: A Single Institution’s Experience. Neurosurgery.

[14] Khalil F., Saemundsson B., Backlund A., Frostell A., Arvidsson L. (2024). Revision and Infection Rate in 728 Shunt-Treated Adult Hydrocephalus Patients—A Single-Center Retrospective Study. World Neurosurg..

[15] Olomo S.A., Obande J.O., Bot G.M., Binitie P.O. (2023). Randomized Trial of Shunt Infection Rates Comparing Intraoperative Vancomycin versus Gentamicin in Ventriculoperitoneal Shunt System Preparation. Egypt. J. Neurosurg..

[16] Gutierrez-Murgas Y., Snowden J.N. (2014). Ventricular Shunt Infections: Immunopathogenesis and Clinical Management. J. Neuroimmunol..

[17] Alqasmi M., Kariri Y.A., Alenazy R., Thabet M., Fallata G., Alqurainy N. (2025). Ventriculoperitoneal Shunt Infections: Causative Pathogens and Associated Outcomes from Multiple Hospitals in Saudi Arabia. J. Clin. Med..

[18] Jaradat A.A., Barbarawi M.M., Jamous M., Jarrar S.M., Daoud S.S., Abdelal A., Haddad S., Alazzam R., Bashyreh M., Ghammaz O. (2026). Acinetobacter Baumannii Ventriculoperitoneal Shunt Infection in the Pediatric Population: Clinical Assessment and Microbiological Profile. World Neurosurg. X.

